# Supplementary material for: Chromothripsis during telomere crisis is independent of NHEJ, and consistent with a replicative origin
Source: Genome Res. 2019 May;29(5):737–49. doi: 10.1101/gr.240705.118 (PMC6499312; doi:10.1101/gr.240705.118)
Supplement: Supplemental Material [file supp_gr.240705.118_Supplemental_file_1.zip › contigs/annotated_contigs/DB53/contig.7.DB53_length_1218_mean_cov_21.9942528736.docx]

**DB53_length_1218_mean_cov_21.9942528736**

```
TGTTTTAAAATTTTTTTTCGGAAACTCTACTTACCTTGGAGGTAATATTTTTTTTAAAAAAAGAGATGAGTAAAACATTTTTGCAGATC
 >chr8:81119898-81120271 - E=3e-212
AGTCTTAGCCATGTCCCTATATCTCAAGAACTTTCCAGGGTGGAACCATGGCTAATGGTTAGAAAATGAGAGGGGGCCTCTCCTCCCAG

AAACCTGCCCTTCTACACCATTCTCAATAGTTAGATGGAGAGTCTGTCAGATGGAAAGAACCACCCAGCATGAGAACAATGGGCCCATC

AAATATCCTTTAAGGGATGACCTTCACTGCAGCCAGCTTCTTCCAGGCCTCCAAGGTAAGAAAAGAGAGCTGGAAGTACTAACATCTAA

GAGAATCCGGTGAT|TCT|CATCAAACCTTATTTTCCTCAGAGGAAGATAATAAT|TT|ACGTATCTCTCATCTCCCTCTCTCTTTCTC
               >chr8:79152219-79152260 - E=1e-12 p=3e-02  >chr8:79152573-79152633 - E
TTTCTTCCCCCCCATGTGCTCAGTTTG|T|CTATGACTATAGAGAACTAGTTTAT|T|GGAATAAAAATAATAATATGGAAATTTGCAC
=4e-24                      >chr8:79152635-79152662 + E=3e-04  >chr8:85468085-8546833
TATAAAGAGCAGGTGAAGGGTAGTGTACATAATTTTCACTGTTTTAGATAATTTGCTTTTGCAATAGAGTCATAAACATTAGAAAGAAC
7 + E=2e-136 p=3e-03
ACCAGAACTTAAAATAACCTGCTCACTTGGGCTTGCCTCTACAGCATGTCTGTATGGTCTGCACTCTCTCCCCGGATTAATTGCTTTCA

TATCATGTACATTTGCAGGAACAGTGTGACA|AATGATAAAAC|CACATCATTACTTCTATGGTATTCCTTGCAATAAAAAAGCATACA
                                >chr8:81118177-81118249 + E=3e-28
TCCTGAAGAAAC|ATCA|CTTGAGCCCAGGAGTTCGACGCTGCAGTGAGCTGTGATTGCTCCACTGCACTCCAGCCTAGGTGATAAAGC
             >chr8:80763442-80763858 + E=3e-230
AAGACCCTGTCTTAAAAAAAAAAGTGGGGCAGTAGAGAAATTCTTTCTTTCTTCTCTCTGTCTCTATCTCTGTCTTTCTTCTGTCCCTC

TCTTCCTCCCCAGGCACATGTTCCAAAGAAATGCCTTGTGAGGACACAGCAAGAAGGCTACTGTCTACTGTCTAGAAGCCAGGAAGACA

GTTTCCACCAGGAACTGAATCAGCTGCCACCATAATATGGAACATTTAGCTTCCAGCACTGTGAGAAAATACAGTTGTGTTGTTAAGCC

ACTCGGTCTACAGTACTTTGCTGTGGCAGCCCAAAGCCAAAGCAAACTCACGCTCTACTAAAATTCACCTCCC
```
